# The Benoni E. Gardner Appeal Case in the Supreme Court of the United States

**Published:** 1872-07

**Authors:** 


### THE BENONI E. GARDNER APPEAL CASE

IN THE

### SUPREME COURT OF THE UNITED STATES.

(From the Dental Cosmos.)

This case was argued on Monday, March 18th.

John A. Foster and Samuel J. Glassey, of New York, counsel of the New York Committee, and B. F. Horwitz, of Baltimore, counsel of Baltimore and Washington Committee, all for appellant; Causten Brown and B. R. Curtis for appellees.

*Official Copy of the Supreme Court Mandate.*

No. 133.

Supreme Court of the United States.

December Term, 1871.

Benoni E. Gardner, appellant, *vs.* The Goodyear Dental Vulcanite Company and Josiah Bacon.

Appeal from the Circuit Court of the United States for the —— District of Rhode Island.

This cause came on to be heard on the transcript of the record from the Circuit Court of the United States for the ——District of Rhode Island, and was argued by counsel. *On consideration whereof*, It is now here ordered, adjudged, and decreed by this Court, that the decree of the said Circuit Court in this cause be and the same is hereby affirmed, with costs and interests until paid, at the same rate per annum that similar decrees bear in the Courts of the State of Rhode Island.

6th May, 1872.

---

The above is a transcript of the decree in this appeal case, as recorded.

The Court adjourned on the 6th day of May, 1872, until October 30th, 1872. On the day of adjournment the Chief Justice made the following announcement:

"In the appeal of Benoni E. Gardner, this Court affirms the decision of the Circuit Court."

The opinion, or decision, of the Supreme Court will not be read until next term. Before that reading its exact wording cannot be anticipated.

We have been informed that two of the judges dissent, but our information does not extend to whether they will so far differ from the others as to offer dissenting opinions.

Our readers will understand that until the opinion, or opinions, are delivered, an exact measure of the extent of this defeat cannot be made.

From the fact that there was a "divided court" upon a case where so many distinct points were litigated, it is not an evidence of insanity to hope that some of these points may have been decided against the Cumming's patent.

The situation is a little ambiguous, yet with the decisions of the lower courts, and the testimony and arguments before us, we may venture to offer the following estimate of its scope and effect, for the information of those dentists who feel like resisting Mr. Bacon's agents and refusing to buy license under the Cummings' patent.

We dismiss the subject of the judgment against Dr. Gardner with costs, etc., and the confirmation of perpetual injunction against him. As regards him personally, it is a contemptuous sham. Fourteen months before the date of the decree here affirmed, Dr. Gardner was made a free licensee for the whole term of the Cummings' patent. And four days after the appeal bond was filed, Josiah Bacon, Treasurer, gave him a written pledge not to hold him for the costs and damages in this suit. This was in fulfillment of the bargain to do so if he would consent to make this appeal.

That which concerns us here is not the sham case between the plaintiff and defendants in error, but the momentous case of the Goodyear Dental Vulcanite Company, of Boston, and Josiah Bacon, Treasurer, against the Dentists of the United States.

The judgment decree, immediately affirmed by the Supreme Court, is that of Mr. Justice Clifford, made on a final hearing of the case in Washington, May, 1870.

The argument for defendant was written by Mr. Foster and spoken by Mr. Glassey, junior member of the firm of Foster & Glassey.

Justice Clifford made his decision in November, 1870.

This decision is not anywhere of record accessible to us. A decree is entered at Providence. It orders a master to assess the damages, etc., and a perpetual injunction to issue against Dr. Gardner.

Mr. John A. Foster is our informant that Judge Clifford refused to write out a new decision, but applied the decision which he had made in the Wetherbee case, and reaffirmed it against Gardner.

We accept the information as true, and conclude that the following points in the decision of the Wetherbee case have most likely been passed upon affirmatively in this denial of the Gardner appeal:

That the Goodyear Dental Vulcanite Company, of Boston, are the owners of John A. Cummings' patent, and the reissues of it.

That this Company, although incorporated as a manufacturing company, has a right to do the business of selling licenses under this patent, thereby manufacturing through their licenses.

That the Cummings' patent is valid notwithstanding the lapses of the applicant, and the irregularities which occurred in its passage through the Patent Office.

That the reissues of the patent are valid, notwithstanding they describe processes which were not known at all when Cummings made his first application.

In brief, that John A. Cummings was the first inventor of the use of hard rubber for making dental plates, and to his assignees belongs the exclusive right to its use for seventeen years, from June 7th, 1864.

Consequently, if any person, who is sued for infringing this patent, shall hereafter attempt to defend on any of these points, the Court, upon the showing of this adjudication, will almost certainly refuse to hear such defense, and immediately grant an injunction, because upon such a showing, a plaintiff should not be put to the cost and delay of a trial.

---

*To the Dentists of the United States:*

**"The evidence in a suit, where only one party is heard, is straighter than a line.**

*Hindu Proverb.*

The announcement of this defeat will convey a sharp pang to very many friends, whose sentiments of justice and equity it will outrage.

There prevails among intelligent citizens in this country a certain common-sense notion that the condition on which an inventor can protect himself is, that he shall procure a patent within the time fixed by law, and thus fairly notify all persons of his claim; and that if he fails, for any reason whatever, to do this, and permits other persons to use and practice his invention or discovery without protest of dissent, he forfeits all right to a lawful patent therein—in legal phrase, dedicates it to the public; and that if he does subsequently get a patent, and undertakes to enforce it, the proof that it was known and in common use publicly for more than two years before the patent was granted, will be a safe and perfect defense against him.

The process and product of the Cummings' patent was in use for eight years before its issue in 1864, and for five or six years of this time it was in large, general, and common use in every State of the Union, more particularly in the State and city where Dr. J. A. Cummings resided and practiced dentistry, and used it himself as others did under a Goodyear license. During all these years he never asserted his right, never notified any person that he had any idea of reviving his claim; while all the time the fact of his having applied for a patent, and failed to obtain one, was also known to many.

Guided by this well-settled and well-grounded belief that such a claim could not be made valid—knowing the law, and that Cummings' patent was granted in violation of it—the dental profession rested safely in the assurance that when, by the expiration of the Goodyear patent, their course became clear to resist it, it would be broken.

The feasibility of this was maintained in the *Dental Cosmos*, by correspondents who had studied and examined Cummings' patent, and was also fully and explicitly enunciated by the able counsel of the Ohio Dental Association, Hon. S. S. Fisher, late Commissioner of Patents, in his written opinion, given in 1868.

In this belief of the profession, in the opinions of our correspondents, and the correctness of the opinion of Mr. Fisher, we had full faith, and still hold that they were each and all sound and right.

We are therefore constrained to assert that this affirmation of the Cummings patent, and the evil effects thereof upon the whole profession of dentistry, through the Gardner appeal case, was positively unnecessary; is largely due to bad counsels and errors of judgment in its management; and is pretty surely the culmination of a deliberately planned and skillfully carried-out imposition.

When the individuals who subsequently organized the Goodyear Dental Vulcanite Company had obtained the Cummings' patent, and attended to its reissues, they strengthened themselves, and greatly weakened the present ability of the dentists to resist them, by buying of the American Hard Rubber Company, of New York, so much of the Nelson Goodyear patent as applied vulcanite or hard rubber to use in dentistry. What Poppenhausen & Co. had found to be a profitless job, Bacon & Co. made congenial and profitable. As a consequence of this purchase, they were able to organize and carry out their system of agents, spies, and enforced licenses; it being conceded that, in the way presented, and for the time it had to run, no successful defense could be made against the Goodyear patent. Here and there resistance to it was attempted—never with success, however; and wiser counsels more generally prevailed, and were submitted to.

Although pushing the collection of license money under the Goodyear patent, and perfecting their organization over the whole country, the Goodyear Dental Vulcanite Company were fully alive to the paramount importance of the Cummings patent. Upon it their hopes of fortune depended. They studied the case and prepared for it by two reissues, and then with consummate boldness commenced an action for infringement against Dr. Wetherbee, of Boston. This suit is commonly known as the Wetherbee case. In it the Bacon party showed great confidence in their own careful and thorough preliminary work. Their counsel carried the prosecution with vigor and ability. The courtesy shown in admitting testimony presented by the defense, in a shape which would have enabled them to exclude it, might be construed into contempt for those who offered it, or the consciousness of great strength—but was in fact an exact appreciation of the legal worthlessness of the evidence on which the dentists relied for proof of priority against Cummings.

Boston was a favorable place for a trial on this patent for all parties. For the dentists it was favorable, because the greater portion of the testimony which was useful in resisting Cummings' claim was attainable there or in its vicinity —for the Bacon party, because, as the centre of manufacturing interests in this country, the courts may be presumed to enter earnestly into the defense of inventors against infringers; because, the spirit of invention being active in that section, the tendency will ever be for one person to use some portion of the invention of another, and rival claims be frequently set up for the same invention. Jurists in these courts become skillful in the law of patents, and whatever may be said or thought to the contrary, their decisions are not often overruled.

The Committee of the dentists associated for defense was capable, worked hard, was tolerably well sustained, had very able counsel, and made a defense which was nearly perfect. We think the Wetherbee case was in every respect honestly managed by the dentists and their counsel, and that, for a first trial, it was very complete. With it for a guide, a case might have been made up for appeal on which the Cummings' patent would have been broken. We quote from the opinion of Mr. Fisher, before alluded to:

"A final decree has not yet been entered in this case, and it may yet be removed, by appeal, to the Supreme Court at Washington.

"If this should be done, it seems to me that the validity of this patent would not be sustained, notwithstanding the opinion of the learned judge. A case has never yet arisen where a patent has been held good which was granted under circumstances similar to those attending the issue of this one to Cummings."

The decision of Judge Clifford was a great surprise to many persons who had given serious attention to the trial. The extreme ground which his honor took in sustaining the issue of Cummings' patent has always been thought to indicate a bias in its favor. An idea was prevalent, which we think has always been maintained among dentists generally, and with many lawyers, that Mr. Justice Clifford's famous opinion in the Wetherbee case would not obtain the support or assent of the other judges.

The proposition to appeal it to the Supreme Court was very popular, and abundant means would have been supplied by those who urged and desired this action. Nevertheless it was wise to refrain until a case was made up, under the care of those who would carry it through, and free from the mistakes which were incidental to a first trial.

This forbearance was predicted on sound judgement, and implied no doubt of the result which might be gained whenever that decisive stroke should be made by a competent hand. There was then no suspicion that a treacherous or collusive case would be devised and carried through the Supreme court of the United States; the Goodyear patent had several years to run; and meanwhile vigilance slept and the enemy wove the net.

The apathy of dentists and dental societies cannot be excused. The dates and interval in which they had time to prepare for an active campaign against the Cummings' patent were again and again announced to them, yet they slumbered supinely throughout the whole period, and gave their enemies full play to concoct this swindle. We do not care to press this. "What is everybody's business is nobody's business,"—this proverb applies to us all, and also excuses us all. However pitiful the result, and however needless it may have been, no one will say to himself, "I am to blame, that I did not work in time to prevent it." On the contrary, too many will be disposed to sling censure around loosely, if only to save themselves from censure.

The Bacon party prosecuted their work industriously during this interval of inactivity. Positive resistants of their claims were brought to Court in several districts, and, with one exception, every suit against individual dentists resulted in an injunction against the victim, and in favor of the Cummings' patent.

Conspicuous among these now, although in its beginning or inception it was almost unnoticed, is the suit against Dr. Benoni E. Gardner, of Providence, R. I. Dr. Gardner was a licensee of John B. Newbrough, and this suit was instituted to make an issue between the Cummings' patent and the patents of Fagan and Newbrough for iodized rubber. This issue affected only a small fraction of the dental profession, and the case owes all its notoriety to the fact that it afterward became the foundation of the collusive artifice practiced upon the United States Supreme Court, and the decree which has just been obtained sustaining the Cummings' patent against the appeal which Dr. Gardner was induced to make.

Dr. J. B. Newbrough, in trying to get the profession to adopt iodized rubber, not only offered more favorable terms than the Goodyear Dental Vulcanite Company, but also pledged himself to defend his licenses; and, in fulfilment of that pledge, he did defend Benoni E. Gardner when a suit was instigated against him. Local counsel, resident in Providence, having been employed to enter an appearance, Dr. N. provided as his counsel Messrs. J. A. Foster and S. D. Law, to make the argument for defendant, on the application for preliminary injunction.

The summons to Gardner bears date Sept. 24th, 1868. A temporary injunction was granted against him Nov. 25th, 1868. Dr. Newbrough did not permit this to lie, but filed an answer Dec. 7th, 1868, and prepared for final hearing upon his main issue, viz., the exemption of iodized rubber from Cummings' claims. The defense, as honestly conducted for Dr. Newbrough, was in reality limited to this, viz., that whereas John A. Cummings had, in his claims and specifications, confined himself to hard rubber vulcanized with sulphur, and named the Goodyear rubber as that which filled the conditions of this specification, and with which only he had worked, the iodized rubber, which was a different compound, was free from the claim of his patent by reason of that difference. Briefly, that iodized rubber was clear of Cummings' claim, because it was a compound of different ingredients from that on which his claim was based.

They began taking testimony April 8th, 1869, and were engaged upon it about three months, when we are brought to a halt by finding that Dr. Newbrough compromised with the Goodyear Dental Vulcanite Company, and ceased to defend this suit. He found himself embarrassed in his efforts to sell and sustain the iodized rubber, and the suits pending against him were tending to adverse decisions. The profession held back from committing themselves to his rubber, and were chary of buying his licenses.

On the other hand, the Goodyear Dental Vulcanite Company had sustained very serious diminution of their income. This was due mainly to the discussion of their impositions by societies, conventions, and dental journals; but they were persuaded to believe that a large portion of their loss was caused by iodized rubber and Dr. Newbrough's opposition.

Through the intervention of a person who had acted previously as an agent for Bacon, but who was then engaged by Dr. N. in exploiting iodized rubber, a compromise settlement was effected. This settlement bears date July 1st, 1869.

According to the terms of it, Dr. N. surrendered his pat.

ents, and became a licensee of the Goodyear Dental Vulcanite Company. Four licensees of Dr. N. were also protected. All these were to receive from the Goodyear Dental Vulcanite Company a free license under the Goodyear and Cummings' patents. The suits against iodized rubber brought under the Goodyear patent, and against its use for dental plates brought under the Cummings' patent, and also the suit to have the Haering patent declared void, were provided for; either by discontinuance or agreements covering their conclusion.

The suit against B. E. Gardner, the defense of which had heretofore been managed by Dr. Newbrough, and the expenses of which had been paid by him, is not named in the agreement ; but that Dr. Gardner was thought of, and his safety and interest considered, is evidenced by the fact that it contracts for a license to be given to him continuously, without cost, on both the Goodyear and Cummings' patents.

Dr. Gardner disclaims having been taken into confidence of his friends or fully informed of his privileges and immunities under this contract. We have his detailed statement in writing, and from it we glean, that Dr. Newbrough came to Providence subsequently to the compromise, informed him that he was free to use vulcanite in his practice, and that the suit was to be continued merely to test priority between the Cummings and Haering patents; that he heard a settlement of accounts between the lawyer Hayes and Dr. Newbrough ; that Mr. Hayes took occasion to say to him (Gardner) privately that up to that time he was safe and clear of all responsibility for costs and damages, and warned him to be careful about signing papers in future.

By the compromise Dr. N. became interested—as also did the friend who managed the settlement—in the increase of revenue of the Goodyear Dental Vulcanite Company, which they all anticipated.

John A. Foster showed us a copy of this agreement, and permitted us at that time to take the names of the signers and witnesses to it, and the date of its execution : the real significance of the document was learned subsequently. We owe our thanks to Mr. Foster for thus starting us on a trail which led to all the documentary proofs of the collusiveness of this case.

Dr. Newbrough asserts, that besides paying Mr. Foster in full for legal services up to the time of this settlement, he recommended him to the Goodyear Dental Vulcanite

Company, and understood that they employed him to carry on one of the suits.

Dr. Gardner says he was informed of the transfer of the management of the suit to Mr. Foster, but had no knowledge that Dr. Newbrough's interest therein was wholly extinguished, nor any suspicion of Mr. Foster having been employed or paid by Bacon. He asserts that, while he knew there had been a general settlement, he supposed that this remained a live issue.

Our readers, by comparing this statement of Dr. Gardner, with what we have shown the contract really was, will discern the intention, which becomes more apparent as we proceed, to delude Dr. Gardner as to the use they were making of him.

Shortly after the settlement, Mr. Foster came to Dr. Newbrough and said that the Goodyear Dental Vulcanite Company wished to continue him (Foster) as counsel for defendant in the Gardner suit, and desired Dr. N. to pay him, and otherwise carry the case along, just as if he was still defending it, and that they (the Goodyear Dental Vulcanite Company) would reimburse and pay back the whole of the cost of such (bogus) defense.

We make this statement wholly on the word and authority of Dr. N., and add, that he claims to have refused, indignantly, to carry out or be a party to any such deception; he also says, that he sold all his interest in the profits of the Goodyear Dental Vulcanite Company, for a small sum, to the friend who negotiated the settlement.

Benoni E. Gardner, being a licensee of J. B. Newbrough, who paid for and carried on his defense when application for a preliminary injunction against him was made by the Goodyear Dental Vulcanite Company, and having been protected by Newbrough in the settlement with a free license,—under both the Goodyear and Cummings' patents, —ceased then any longer to be a veritable defendant, although his name was retained in the case; John B. Newbrough, being also a licensee of the Goodyear Dental Vulcanite Company, and having disposed of his iodized rubber, had no interest in the defense of this case after July 1st, 1869, although, before that date, he was the defendant in fact.

After the settlement with Dr. Newbrough, somebody went to Providence and obtained from the local counsel there, who had entered appearance for Dr. Gardner, an assign ment of the case to J. A. Foster, giving him control thereof as leading counsel of record, subsequently to which the strategic game of making up a case for appeal to the United States Supreme Court went on, beginning in August, 1869, and ending with decree and appeal in November, 1870.

A careful examination of the record begets in our mind the conclusion that there never was a sincere intention to make a good or perfect defense. Stipulation after stipulation was made—in each instance to favor and cheapen the process of prosecution to the Bacon party.

The testimony being all in Sept 13th, 1869, there ensued Nov. 20th, 1869, the stipulation No. 2 of Record: "It is hereby stipulated and agreed that this cause may be heard on pleadings and proofs by Mr. Justice Clifford, sitting at Chambers at Washington City, and that a decree may be made by him thereon at said city as if made in Rhode Island."

What attorney conducting a true defense for a genuine defendant, would have consented to such a stipulation as that?

Can a dentist be found who believes that consent to go out of the bounds of the circuit to plead before the judge who had decided the Wetherbee case so pronouncedly in favor of the patent, was ever given by a counsel who sought to win the case?—or that Mr. Foster did not know that he thereby bargained away all chance of success?

For the work done upon this case after July, 1869, viz., taking testimony before Commissioners during thirteen months, and making up the case for appeal, we have been told that Mr. Foster was paid by the Goodyear Dental Vulcanite Company, and that for a "spread-eagle" speech before Judge Clifford, and other services, Mr. Glassey was also paid by the Company. We ourselves believe the information, but the difficulty of making proof where the witnesses are nearly all implicated is such that we ask our readers to class it as a rumor.

The examination of witnesses, conducted wholly in New York, before a United States Commissioner, was concluded in September, 1869. The case, on final hearing, was argued before Judge Clifford, at Washington, in May, 1870. His decision was probably oral. The Record exhibits only a Decree, September 8th, 1870, referring cause to a master. Master to take and state to the Court an account of gains and profits, etc. Injunction to be issued against the defendant.

The date, September 8th, 1870, is an eventful one. The Goodyear Dental Vulcanite Company had several things to get done, and decided to put them all through in one day. See the Record:

September 8th, 1870. Decree, referring cause to master, etc.

September 8th, 1870. Parties appeared with their counsel and agreed that the master should report the sum of seventy-five dollars as the gain and profit.

September 8th, 1870. Final decree. Upon the coming in of the report, Benoni E. Gardner was *perpetually enjoined* from making, etc.

September 8th, 1870. Benoni E. Gardner appealed the case to the United States Supreme Court. Appeal allowed in open court, bond to be filed within ten days.

Of these four items, the first is probably a genuine result and date. *The parties*, being Josiah Bacon & Co., were fully informed of the day when that decree would be entered, and entirely unanimous about what else was to be done.

The written statement and conversation of Dr. Gardner serve us admirably at this stage of our history. Dr. G. says of the second item, that the only counsel whom he saw was Mr. Jabez Holmes, attorney for Josiah Bacon, who appeared to him on that day, and told him that he represented *the parties*. Some account was to be made out. He went with Mr. Holmes to some office. In reply to questions, he told them that he had no accounts by which to make up a statement of his profit on rubber-work. Had been told that the use of it was free to him, and therefore exercised no care about it. Finally, they computed from the number of cases he remembered making, and agreed to call it seventy-five dollars.

Then Mr. Jabez Holmes told him that *the parties* had agreed to appeal the case. He avers that this was the first and only intimation of an appeal which he had ever heard, and that the absurdity of the thing was so apparent as to cause him to remind Mr. Holmes that he (Mr. H.) was attorney for the plaintiff who had just gained the case, and therefore could not quite understand how the party winning could reconcile themselves to solicit the defendant to appeal.

Mr. Holmes replied that it was all agreed upon between *the parties*, and a Supreme Court decision was required to settle whether Cummings or Haering was ahead in making this invention.

Dr. Gardner is quite certain he was told this for a reason to quiet his surprise, and he believed it. He never had any other idea of the appeal, and was satisfied with that part of the story. If his friend, Dr. Newbrough, had come thus to him, he would have consented without any condition ; but as he was then dealing with strangers, he recalled the warning of Mr. Hayes, and demanded some guarantee that he should be kept safe from the present judgment and future costs. Mr. Holmes offered, as attorney for the Goodyear Dental Vulcanite Company, to give him such a guarantee. But Dr. Gardner refused to accept that, and required to have it in "Bacon's own handwriting." Upon receiving Mr. Holmes' promise that such a safeguard should be forthcoming, he executed the papers which were prepared, but explicitly denies having furnished any security on the bond ; this bond is signed

"Benoni E. Gardner,"
"Henry W. Gardner."

The bond was filed Sept. 17th, 1870. The doctor denies having any knowledge of Gardner No. 2.

A different witness attests the signature of Henry W. Gardner. As our Dr. B. E. Gardner does not mention a second interview with Mr. Holmes, it is probable that the security Gardner was hunted up during the interval of nine days which elapsed before the filing. This is not worth mentioning, except to show that every detail of this appeal was managed and provided by the Goodyear Dental Vulcanite Company without any care to conceal their complicity.

Dr. Gardner subsequently received a letter from Josiah Bacon, Treasurer, written wholly in the hand of that gentleman, which confirmed the guarantee of Mr. Holmes.

This document, published in Appendix, sets at rest every doubt about the collusive character of the appeal. Taken in connection with the contract of Dr. Newbrough, the proof is complete. In this letter the unblushing Josiah stands forth as both plaintiff and defendant in his own person ; for the interest which he saddles upon Dr. Newbrough had been assigned by Newbrough to him fourteen months before!

Why did they intrust this business solely to their own attorney? Was Dr. Newbrough no longer available when dirty work was to be done? Could they not trust Mr. Foster to act a part, as counsel for the defense, in so important a scene as this of the appeal? Was it merely a fit of economy, or had they grown reckless and careless of all decency or propriety, that they should rush four important entries upon the record in one day, with only their own attorney present?

We suspect—but it is only a suspicion—that Mr. Foster was out of favor at that time in the Bacon councils. Certain it is that, a short time after this appeal was made, he was either indiscreet or revengeful; for in December, 1870, he came to Philadelphia as counsel and advocate of the Ready Rubber humbug, trying to get us to aid in inducing dentists to take licenses or rights to use Ready Rubber, and stated in the presence of this writer, and two other reliable witnesses, plainly, that of his own certain knowledge the B. E. Gardner appeal case was a collusive one, made up to gain a decision in favor of the Cummings patent in the United States Supreme Court. His exact words were: "Both sides made up by Causten Brown, and the best points against the Cummings patent left out." (Causten Brown, Esq., is the working counsel of the Goodyear Dental Vulcanite Company.)

Will our readers accept John A. Foster, Esq., this eminent champion of dentists' rights, (whom they have seen lauded and indorsed by committees and societies, with requests for dental journals to publish their praises of him,) as a competent witness to the character and intention of this celebrated B. E. Gardner appeal case? Does he, who carried it through all its stages up to final hearing and decree, know for whom it was got up—and why?

In December, 1870, he uttered this opinion and denunciation of it. He repeated it more than once—on more than one day—to and before several persons; not casually, but circumstantially, with statements of detail, and arguments, and assertions to show that the Cummings patent was sure to be sustained—on a case thus prepared—in the court of last resort, and that the only way open for escape was the plastic Ready Rubber way, of which he was the able advocate. In order to put this "Ready Rubber" humbug on the dentists, he thought it was necessary to exhibit it as a refuge from the Cummings patent—hence this revelation, which we have since confirmed by irrefutable proofs.

The next phase in this suit which claims attention is when this versatile genius, having made a case for a defendant who must have been the plaintiff—for whether we consider him as working for and paid by Dr. Gardner, Dr. Newbrough, or the Goodyear Dental Vulcanite Company, is no difference (after July 1st, 1869, all these parties, as indicated by facts stated, were in the same interest;) having made this case in the way we have shown; having been managed and paid by somebody interested in losing it, (the proof of which, contained in Dr. Newbrough's contract of settlement, he held a copy of in his own desk;) having denounced it as a collusive fraud, when trying to stick Ready Rubber on the dentists—Mr. Foster now conceived the brilliant idea of enhancing his gains by dealing with parties who had a real interest in the defense, and getting more pay from them.

The dentists as a body knew nothing at all of this case. As the time for its hearing approached, it has been stated that a person in authority in the Goodyear Dental Vulcanite Company came from Boston to New York, and said—we quote verbatim as related—"It is going on too flat; it is too thin; we must have the appearance of resistance against us. Stir up the dentists; get them to give their notes; we must have a show of genuine defense." Whether carrying out the orders thus indicated, or working for his personal profit, the coveted appearance of resistance was created, and from this time forth Mr. F. has apparently worked as though he was the sincere advocate of a genuine defense.

At a meeting of dentists held in New York, December 23d, 1871, we had the pleasure to hear Mr. Foster, in a very long and artful speech, set forth that this appeal case was genuine; that B. E. Gardner was too poor to carry it through; that his defense had in it the certain elements of destruction to the Cummings' patent; that he had worked hard and long on it, from pure love of the task of defeating a gigantic fraud.

He so far impressed this audience that a committee was appointed. Contributions in money and notes were solicited. The cash first asked for, five hundred dollars, was paid; then five hundred dollars more was demanded and that also was paid.

As Mr. Foster was about to retire from this meeting, he stopped to remark that it might be deemed desirable, by organizations of dentists in other cities, to associate their own counsel with him. He said that he had met the counsel of S. S. White in Philadelphia, and found him well informed on the points which were involved in the contest with the Cummings patent, and that if the dentists of Philadelphia should decide to employ associate counsel, he would be glad to have him on the case.

If the question, why we did not denounce him then and there, arises in the mind of any reader, it is answered by the explanation that we attended the meeting only to listen and learn. We had not joined in the call, nor participated in the movement. Being, however, requested, toward the close of the meeting, to speak, we did feel compelled to express disapproval of the speculation proposed, viz., that for a service which a business man would procure first-class counsel to do, and get well done, for the ordinary fees (five thousand dollars would be a liberal sum to appropriate,) dentists were here asked and expected to give, in ready cash, a respectable fee and provision for expenses, and in addition thereto a contingent note of fifty dollars. These notes, if given by one-half the dentists, would amount to three hundred and twenty-five thousand dollars.

This was not the sort of action which we felt like recommending. If dentists had need of lawyers, they were able and forehanded enough to employ them as liberal business men would, and not be thus drawn into a speculation which subjected them to a prospective payment amounting to fifty times the value of the services to be rendered.

It is asserted that there were at that meeting three or more dentists furnished with a letter from one who knew and could prove what he wrote, charging that the case was collusive, and that J. A. Foster knew it to be so. That letter was returned to the writer of it, with the statement that the meeting was too enthusiastic for going on with the appeal —too full of friends to Foster for them to venture to check it.

This is cited to evidence how futile any attempt of an outsider would have been to divert the course of this meeting from the original intention of those who controlled it.

One gentleman, however, had the courage to say to Mr. Foster that it had been reported that he (Foster) had recently received money from the Goodyear Dental Vulcanite Company, and that he would like to be informed about it.

With a smile that was bland, Mr. Foster replied, "If I have received any money from them, I would like to know where it is." Mr. Glassey, his junior partner, undertook to explain to the meeting that two thousand eight hundred dollars had been paid to them by the Company, for services rendered to Dr. Newbrough, which the Company had agreed to pay when they made their settlement.

This explanation of Mr. Glassey is not sustained by our examination of the agreement. It does not contain any stipulation that the Company should pay these expenses. Dr. John B. Newbrough asserts that each party was to pay their own costs and expenses; and he offers to prove that he did pay Mr. Foster in full, for all his services in the defense of Dr. Gardner. Mr. Glassey's admission of the receipt of money from the Company, by both Mr. Foster and himself, is, therefore left standing,—by reason of his explanation being severed from it,—as a simple confession of the charge.

Having returned to Philadelphia, an early opportunity was made to reconsider Mr. Foster's statements of December, 1870, and compare them with those now made December, 1871. The speech and points of 1871 were repeated to one, and then to the other, of those who had heard his conversation in 1870. The first exclaimed "He is a scoundrel; he told us that it was a collusive case, both sides of which belonged to Bacon, and sure to be affirmed by the Supreme Court." The other said, "He is a shyster; did he not tell us a year ago that this was a collusive case, both sides made up by Causten Brown, with the best points against the Cummings' patent left out, and sure to be affirmed by the Supreme Court?"

To the counsel, referred to by Mr. Foster as being well informed upon the subject, we expressed unwillingness that so important a case, involving such serious results, should be presented to the Supreme Court by the person who had made these irreconcilable statements in regard to it. If the assertion, that the case had been made up in the interest of the Goodyear Dental Vulcanite Company, had in it a possibility of being true, it was rash and unsafe to intrust it to one who had aided, even unwittingly, in that deceit.

Yielding to our urgency, this gentleman consented to act as associate counsel, upon the conditions that he should be furnished with the Supreme Court record of the case, and the brief and points of the counsel for defense, giving Mr. Foster his choice to open or close the argument.

While these inquiries and arrangements were being made, a desultory correspondence was going on with some members of the New York Executive committee. "Out of the abundance of the heart the mouth speaketh,"—so a hint of doubt in the integrity of the appeal, and also in Mr. Foster, escaped us in one of the notes. This elicited an earnest request for further information from the person to whom it was written.

Fortified, as we now were, by the certainty that two friends could testify to Mr. Foster's statements of 1870, and also by being able to offer an associate counsel of integrity and recognized ability, there was no hesitation on our part, and an arrangement was made to meet and talk it over in New York. At this meeting it was immediately discovered that, to the employment of Col. Foster and the maintenance of the appeal, the Committee had positively pledged themselves.

They nevertheless listened to a full account of the contradictory nature of Dr. Foster's statements in 1870, at Philadelphia, as compared with the opinions he now held and uttered. We were not at that time in possession of the proofs, which have since been so kindly and liberally supplied by Dr. Newbrough, Dr. Gardner, and several other persons, but enough was known to make us anxious to guard against treachery and the faulty preparation of the case. A letter, addressed to Dr. Dwinelle, was read. This letter related a conversation with Mr. Josiah Bacon, and told how he had boasted that the Gardner appeal was fixed so that they could not fail to win it, because there was to be no real defense. It was conceded that the one sure way to act was, by adding to the defense counsel of unimpeachable character. Our proposal to furnish such an associate was accepted. The hopes and fears which governed us are told in the following extract from the Committee, officially communicated and published in the *Dental Cosmos*, February number, page 113:

"The Committee desire, on behalf of the dentists, to acknowledge the liberality and the fraternal spirit with which Dr. S. S. White is aiding in the present exigency. While he carefully *disclaims any indorsement* of the *Dr. B. E. Gardner appeal*, he has contributed generously to the first amount, which it was necessary to raise immediately, because he wishes to aid us in doing all that it is possible for us to do under the circumstances.

"The brief period between the appointment of the Committee and the calling of the case for trial (which is likely to be reached by February 15th,) made it impossible for societies at a distance to so mature their plans that they could employ additional counsel in time to permit them to study the case.

"In this emergency Dr. S. S. White, having been informed that Hon. S. S. Fisher, who had studied the case thoroughly while working as chief counsel for the dentists of the West, has taken a retainer from Bacon to go on the shelf, and fearing that the case might be lost for want of proper watchfulness, and *hoping to avoid the hearing if it should be found that it could not be properly presented*, while utterly declining to influence any other person to aid or subscribe to the maintenance of this defense, proposed to the Committee to employ, at his own personal cost, counsel of eminent ability, to be associated with Col. Foster.

"This offer the Committee thankfully accepted. Calling upon Col. Foster with Dr. W., he offered an eminent practitioner of Philadelphia as associate counsel, who was promptly and fully accepted as such by him.

"W. A. Bronson, *Sec. of Committee.*"

This prompt acceptance of an associate by Mr. Foster was very grateful to some members of the Committee, who had anticipated reluctance or refusal from him. Their confidence in the sincerity of his acceptance is abundantly indicated by the fact that they published it. We had then no idea that he could venture to evade and repudiate such an agreement.

The understanding with the Committee was, that joining thus in their action was not to be construed into any relinquishment of our suspicions.

Shortly after this, we recited these facts, fully and unreservedly, to Dr. Hunt, of the Washington and Baltimore Committee; Prof. J. Taft, of Cincinnati; Dr. John McCalla, President of the Pennsylvania State Convention; Dr. W. N. Morrison, of St. Louis, and several others; never hesitating to speak plainly to all who represented committees or societies, although it was utterly impossible to make circumstantial replies to the many friends who wrote to us during these weeks for information on the "appeal case."

Cotemporaneously with the official statement quoted above, the New York Committee offered for publication in the *Dental Cosmos* a "communication" written by Mr. Foster. The article, besides other vicious misstatements, had this sentence in it: "The case of the Goodyear Dental Vulcanite Company *vs.* Benoni E. Gardner *has been selected by the dental profession as a test case.*"

We refused to print this as an anonymous article, and only permitted it to appear when they indorsed and requested its publication.

We knew that the statement above italicized was untrue, and we believed that it was designed to close the mouths of dentists when the case should be lost.

We reprint this unqualified approval of Mr. Foster's article by the Committee:

"The Committee of the Dentists of New York and Brooklyn take pleasure in acknowledging the receipt of the following communication for publication in the *Dental Cosmos*, and which is, for the most part, made up from the records of the cases now before the United States Supreme Court.

"It is very timely and straightforward in its statements, and is laid before the profession in the hope that it may, if possible, increase the energy and strengthen the hope,—we trust well founded,—in the efforts now being so widely made to free ourselves from an unjust and onerous tax."

That, after our refusal, the Committee were willing to allow Mr. Foster to write in their names, could not justify us in giving such falsities even a constructive indorsement. We therefore published in the same number (page 116, February *Dental Cosmos*) a general disclaimer of personal responsibility for any statements contained in our printed correspondence or communications.

We did this to avoid adding to the number of those who, confiding in false statements that the Cummings' patent was about to sustain a real attack likely to break it, would, because of that belief, resume the use of rubber; Bacon meanwhile lying mute, with claws drawn in, but watchful and eager to pounce upon them the moment the validity of the patent should be affirmed.

How many dentists are this day willing to say they ever had a thought of "selecting" the Gardner case? By just that number our disclaimer will be censured, and the rest must sanction or justify it.

Asking the reader to remember the conditions on which our counsel had consented to act, we resume the narrative:—

The record was not sent by Mr. Foster as promised; after days of unnecessary delay, an imperfect copy (lacking sixteen pages) was received. A request was made for the missing pages, to which no attention was paid. Two weeks later, the request was repeated; in reply to this Mr. Foster wrote, March 2nd, refusing to supply the record until "*after the argument*," and stating—

"At the request of the New York Committee, and on the suggestion of the profession in Baltimore and Washington, I have associated Mr. Orville Horwitz with me, who, with Mr. Glassey, will assist me in the argument." Thus refusing, March 2d, the assistance of the counsel which he had accepted January 31st.

The relief experienced at this severance of all connection with, or responsibility for, this most impudent fraud upon our friends,—many of them life long associates and co-workers,—served greatly to tone the indignation which Mr. Foster's rudeness and evasion of his agreement naturally awakened.

We must here set out the conditions which made this action of Mr. Foster exceedingly prejudicial to the success of the appeal.

The case, as made up, was cunningly devised to touch upon nearly every point which it was desirable to clinch by the decision.

On prior invention in this and foreign countries, the answer was rich in promise, but the proof was utterly inadequate to sustain it.

Haerring's testimony seems to have been put in wantonly to condemn him as a witness forever; for, by the contract with Newbrough, the Haering patent was to be assigned to them, in the event of its validity being sustained.

On cheoplasty the testimony was considerable, and well calculated to undermine a great portion of Cummings' claim to have invented any of the process of making a rubber case; the ANSWER, however, does not fully set it out. It was, therefore, very doubtful if the weight of this testimony could be availed of in the argument.

The answer denies broadlv that the issue of the patent in 1864 was legal. It is also explicit on irregularities of the Commissioner, who had presumed to override the decisions of his predecessor, and condoned the failure of Cummings to complete his application within the legal limit, whereby it was outlawed or dead.

The testimony included a copy of the file wrapper and contents of the case, as found in the Patent Office. On this there was a chance that a good patent lawyer might base a fatal attack upon the title at some point, because it is so flimsy and rotten. Permit a brief statement of it. In 1853 J. A. Cummings filed a caveat. In 1855 he applied for a patent.

So ignorant were both Mr. Cummings and his patent solicitor of the general subject on which he professed to be an inventor, that in his first application he included gutta-percha and claimed it as broadly as vulcanite, not knowing that it had been used long before. After several amendments of it, his application, and all the appeals which he made, were finally rejected in 1857. Two years afterward, having submitted to the rejection without trying the further appeals which by law he might still have claimed, he asked permission to withdraw his application and drawings from the Patent Office. This was refused, because it was a REJECTED application, and, as such, the papers were by law or rule required to be kept in the archives of the Patent Office.

The law of 1861, which changed the patent term from fourteen to seventeen years, and abolished the system of seven-years extensions, contained the following clause:

> "All applications for patents shall be completed and pre pared for examination within two years after the filing of the petition, and in default thereof they shall be *regarded* as *abandoned* by the *parties thereto*, unless it be shown, to the satisfaction of the Commissioner of Patents, that such delay was unavoidable; *and all applications now pending shall be treated as if filed after the passage of this Act.*"
>
> 2d March, 1861.
> 12 *Stat.*, 247.

It was three years after the passage of this act—viz., in 1864—when Cummings' application for a patent was renewed. Upon his consideration of the application, the

Commissioner not only condoned, without notice or petition, this lapse of one year, by which, even under this act, it was outlawed, but entertained and passed upon as *pending* a case which his predecessor, in 1859, had classed as *rejected.* It surely could not be both REJECTED and PENDING. If it really was "PENDING," Cummings would not have been permitted to kill it in 1859 by withdrawal of the papers. If "*rejected*" was true, then it remained true always, and either way it was wrong to entertain it as a pending application under the law of 1861, as by the words of that act it should have been "regarded as abandoned." (See quotation above.)

On such a flimsy, galvanized fraud of an application the Cummings patent was granted, and this defective title is the sole claim on which the Goodyear Dental Vulcanite Company can raid and harry the dentists from May 6th, 1872, until June 7th, 1881.

Were we not justified in hoping that a good, sound, patent lawyer, of great practice and in good standing with the Supreme Court, might, if working earnestly, do something toward breaking such a patent title as that? We believe there were just grounds for such a hope. We believe now (and so do many others) that it was because the real owners of this appeal feared this counsel of ours—feared to risk the danger of having him let in even on so wretchedly prepared a defense—that he was denied.

Yet we were silent, except to the Executive Committee of New York. From them, as the responsible employers of Mr. Foster, we obtained their written certification that his action in dropping our counsel was done without their knowledge or consent. We claim that our silence was discreet. It was coupled also with the acceptance of Mr. Horwitz. He was certainly honest, having been selected by dentists. He might be capable, too, although he had no public reputation or notoriety as a patent lawyer by which to judge of him. We dared not raise a finger or say a word to warn or notify the large body of our friends, over whom we saw this danger and treachery impending.

And so this case, concocted purposely, as we firmly believe, to procure a decision from the Supreme Court of the United States in favor of the Cummings patent, had to go on, trusted to a leading counsel who, it was feared by many, was neither safe nor sincere, assisted by his partner, who was admitted to practice in that court on the morning of the hearing, on the motion of Causten Brown, (they had no fear of Mr. Glassey), and one true man, brought in with so short a time for preparation that in his argument he felt bound to say to the judges, "I am not as familiar with the record as I ought to be, I admit." Not one of the three, it is believed, had ever before appeared in any patent case before the United States Supreme Court.

Gentlemen of the dental profession, have we shown that we had no part in bringing on this defeat? that we did all we knew to save it from being a disaster and to convert it into a triumph? Have we, at any time, by any utterance either spoken or printed in the *Dental Cosmos*, disheartened those who thought it their duty to work in this case? Have we induced any one to give any money toward it, or in any way aided to increase the number of dupes to it, or spread the delusion that it was ever meant by its managers to hurt the Cummings patent.

Some of those who were controlling and guiding the case to the hearing, were as cognizant of the true character of it as we could make them. Their mental vision was warped. They were intoxicated with the prospect and hope of success, and nothing but the bitter conclusion would sober them wisely. Others who were working in it were true and earnest men, giving much time to the collection of money and notes, though but little, if any, to the study of the case. We refrained from publicly exposing its character, because we had no right to stop their work; for with the day of the hearing so near, it would be folly to impair their force or weaken their standing. We gave money as liberally as any one did. We printed their notices, appeals and statements, with one single disclaimer; and when the crisis approached, having personal knowledge that over fifteen thousand dollars were pledged if the patent should be broken, hoping that the abundant reward which he was sure to reap might stimulate him to work sincerely for these his last clients in it, S. S. White, in a note addressed to Mr. Foster and delivered to him on the morning of the hearing, offered to give him five hundred dollars in addition if he won the case.

The wide-spread concern wich is felt in it by a large portion of the profession, moves us to make so long and detailed a statement of this case.

Having carefully gathered the proofs which sustain all we say, our aim in this article is not only to vindicate ourselves, but also to set up the workers in this case in such clear light that their real relations thereto shall be incontestably manifest.

To know correctly and truthfully the procession of persons and events which have moved to this result, will enable our readers to judge of it soundly, and to discriminate between real enemies, rash or imprudent friends, and those sincere workers who chose to risk temporary censure and obloquy, rather than to aid in precipitating others into disaster.

Besides many things of interest omitted in order to relieve our readers from too lengthy a recital, we have refrained from every kind of personal censure upon those well-meaning dentists who thought they saw in this case an opportunity to redeem themselves and the profession from a galling imposition ; and who, thinking so, blamed as luke-warm or traitorous, those who because they knew more and saw more, could not share their faith or enthusiasm. Failing to comprehend all that passed before, them and blinded by Mr. Foster's statements, some of these persons have not hesitated to represent us as being antagonistic both to them and the case itself. Candid and just men will accept this veracious narrative as correcting all misrepresentations, and the calumny will be short-lived. For these censorious friends, who were yet true haters of the injustice of the Cummings' patent, this defeat is punishment only too severe. They feel personally worsted.

What all this brings us to is the present relation of dentists to vulcanite work and the patent which controls it. The Goodyear patent expired May 6th, 1872. On that day this decision was announced, which gives to the Cummings patent a standing in court which deprives individual dentists of any hopeful chance of resisting it.

There is naturally due to a decision of the Supreme Court of the United States a certain weight and effect which this one will fail to secure permanently. We charge that the parties by whom this case has been made up have committed a deliberate contempt upon this highest tribunal of our country ; that they purchased this case, so as to control it ; that since its purchase, they have directed both sides of it, for the express purpose of gaining a decision rapidly and safely. Reckless of the dignity of the courts thruogh which they have worked : reckless of the interests of thousands of dentists whom they plotted to entrap in meshes from which they now represent that there can be no escape; reckless of the professional honor of their own ablest advocate; reckless of everything but the success of their plans, and their selfish greed for a harvest of plunder.

We charge that in this case, since the settlement of the Goodyear Dental Vulcanite Company with John B. Newbrough, July, 1869, there has been no veritable *plaintiff*, because they were bound by that settlement to *give* a full license to the man whom they are, in this suit, pretending to sue for infringement. There has been no veritable *defendant*, because B. E. Gardner was licensed, and protection for him secured in Newbrough's contract.

We charge that this case has, ever since July, 1869, been collusive, and that it is a fraud upon the courts, and upon those against whom this decision is now sought to be enforced, and has never had any color of verity, except that which it obtained through the misguided efforts of those dentists who aided in giving it that semblance by their action just previous to and at the hearing.

Such a decision—a decision so obtained—is not entitled to, and will never receive, the deference and respect of intelligent, law-abiding, and equity-loving citizens.

The proofs of collusion and fraud, which we have gathered and hold, are safe beyond the possibility of concealment or purchase. They are ready to aid whatever resistant action the dentists of the United States may decide upon or be advised to take; whether that shall be to seek a rehearing of this badly-prepared case, or to claim a fair hearing on a new one.

Be sure, that when presented, the court will listen to them, and that this decree of May 6th, 1872, will NOT be FINAL. We advise our friends not to believe it, not to submit to it, nor to think of it as FINAL. Dentists should cease the use of rubber, and refuse to take or pay for licenses. Half-way measures will not do now. The refusal must be absolute, and it must be general. In the present crisis the Russian policy, strictly carried out, is the only one which will defeat the enemy and secure our own future self-respect. *Devastate the country through which they must march, and burn Moscow.* Let us test the question whether there is in this great body of educated men enough self-denial to make this course a succsss.

If this feeling and determination is established in the minds of individuals, it will be communicated from one to the other, extend to local meetings and conventions, and become accepted and recorded as the decision of these primaries.

Out of these a league may be formed, and a national organization set up, which will exert an influence strong enough to draw in the wavering, and those who have calculated upon making profit out of the abnegation of their brethren.

Ultimately, with a careful selection of leaders, wise forethought· the best and ablest counsel in all the land, and the abundant means which a very small contribution of a host of members will aggregate, this Goodyear Dental Vulcanite Company shall suffer first the extinction of their revenue, and then a trial, which will extinguish the title of their Cummings patent.

SAMUEL S. WHITE.

### APPENDIX.

**EXTRACTS FROM THE CONTRACT OR AGREEMENT OF JOHN B. NEWBROUGH. ORIGINAL IN THE POSSESSION OF S. S. WHITE.**

This Agreement, made and entered into this first day of July, A. D. 1869, by and between John B. Newbrough and Henry E. Fickett, of the city, and county, and State of New York, of the one part, and the Goodyear Dental Vulcanite Co., a corporation created under the laws of said State, of the other part, witnesseth:

Whereas, Letters Patent of the United States have heretofore been issued to the said John B. Newbrough, and to one E. Fagan, as follows: No. 70,250, issued October 29, 1867; No. 73,545, issued January 21, 1868; No. 73,916, issued January 28, 1868; No. 73,917, issued January 28, 1868; all being for improvements in treating caoutchouc, gutta-percha, and similar gums, and in the manufacture of articles therefrom; and

Whereas, By assignment duly made and executed, the said Fagan has sold, assigned and transferred all his right, title and interest in and to said several Letters Patent, and the inventions described in and secured by such patents, to the said John B. Newbrough, of New York; and

Whereas, The said Goodyear Dental Vulcanite Company is desirous of terminating the litigation upon the question whether the compositions in said Letters Patent described, and the material hereinafter mentioned called Oilite, are infringements upon the patents of Nelson Goodyear, owned by said company; and

Whereas, Said company, without in any way admitting the validity of said Newbrough and Fagan Letters Patent, or that the compositions therein described are not infringements as aforesaid, or that the material called Oilite is not an infringement as aforesaid, have requested the said Newbrough to assign the said Newbrough and Fagan patents above mentioned, so far as they relate to, or are applicable for, use in the manufacture of artificial plates for dental purposes, unto Henry Redfield, of New York, in the State of New York.

*Now this Indenture witnesseth,* That the said John B. Newbrough, for and in consideration of the sum of one dollar to him in hand paid, and of the covenants and agreements hereinafter contained, and to be performed by the said Goodyear Dental Vulcanite Company, has agreed to sell, assign and transfer, and has by an instrument of even date herewith, sold, assigned and transferred to Henry Redfield, his heirs and assigns, the said several Letters Patent above mentioned, and the inventions described therein, so far as the same relate to, or are capable of being used for, dental purposes, or for the manufacture of artificial plates or other structures for setting artificial teeth, or other uses connected with dentistry, but for no other use or purpose.

To have, and hold, and enjoy the said patents and inventions for such uses and purposes, but not otherwise, during the existence of such Letters Patent, within the United States, as fully and entirely as the same could have been held and enjoyed by the said Newbrough, had this sale and assignment not been made; and

WHEREAS, Letters patent of the United States were, on the nineteenth day of January, A. D. 1869, issued to Robert Haering, numbered 85,997, for a "New Mode for Mounting Artificial Teeth," which said Letters Patent were on the ——— day of ——— assigned to said Newbrough, who is now the owner thereof, which said Letters Patent are believed by said company to be illegal and invalid, although this cannot be known with certainty until the question is settled by a judicial decision. It is therefore agreed that if said last mentioned Letters Patent shall be decided to be legal, then the said Newbrough shall, by a good and sufficient conveyance, convey and assign said Letters Patent, and invention, and all rights thereby created, to said company, or to such person as they may appoint or direct; such assignment, however, shall not take away or impair the rights of any persons to whom said Newbrough has given licenses to use said Haering patents before this date.

The said company, for itself, its successors, and assigns, further covenants and agrees to give and grant to such persons as may be designated or nominated by Mr. Henry E. Fickett, five dental licenses to make and use hardened rubber, gutta-percha, and similar gums, for dental purposes, such licenses to be granted or renewed so long as said company, its successors, or assigns, shall continue to grant similar licenses to others. * *

It is agreed that the suits now pending between the company and said Newbrough shall be disposed of as follows: The suit upon the patent of Nelson Goodyear, in which the question of the infringement by the use of iodized rubber is raised, shall be dismissed without prejudice or costs to either party. In the suit against said Newbrough, for infringing the Cummings patent, a final judgment for the complainant shall be entered, with nominal damages. The suit for the repeal or setting aside the Haering patent shall be left to the Court for their decision, whether the Haering patent is legal and valid or not.

The licensees named by said H. E. Ficket, and made a part of this contract of settlement, are John B. Newbrough, New York; ———, of New York; ———, of Brooklyn; B. E. Gardner, of Providence; and ———, of Cleveland, Ohio. In case of death of any of these parties, no other is to take the place. But of the persons here named, no sum of license fee shall be demanded which shall exceed in amount the sum accredited to each, during the continuance of the patents.

CHAS. E. HILL, witness. — J. B. NEWBROUGH, [L. S.]

JOHN L. COBB, witness. — HENRY E. FICKETT, [L. S.]

JOHN H. CHEEVER, Pres. G. D. V. Co. [L. S.]

JOSIAH BACON, Treas. G. D. V. Co. [L. S.]

GOODYEAR DENTAL VULCANITE Co. by J. H. CHEEVER, Pres. [L. S.]

---

COPY OF A LETTER FROM JOSIAH BACON, TREASURER, TO BENONI E. GARDNER. ORIGINAL IN POSSESSION OF S. S. WHITE.

GOODYEAR DENTAL VULCANITE Co. JOSIAH BACON, TREASURER.
No. 5 Temple Place, Boston, Mass.
No. 38 Park Row, New York.

BOSTON, September 21st, 1870.

DR. BENONI E. GARDNER, Providence R. I.

DEAR SIR,—I have had some conversation with Jabez Holmes, Esq , my attorney, in regard to the appeal of the suit decided against you, and your liability for the cost thereof.

I will therefore say to you, frankly, that though the suit is against you in name, yet Dr John B. Newbrough is the real defendant in the case, and is defending it, and you are only the nominal defendant. I do *not*, therefore, hold you responsible for the cost, etc., but I do most distinctly hold said Newbrough responsible for both costs and damages to the uttermost dollar.

The suit was appealed by Newbrough in your name, as both parties thereto wished the matter passed upon by a court of final jurisdiction, as the patents of Newbrough are either paramount or subordinate to ours, and the question must be fully decided.

In regard to your own position, permit me also to add, that you are now under perpetual injunction by the United States Courts, forbiding you the use of any rubber whatever, either iodized or any other kind, and any use thereof by you will subject you to a heavy punishment by the court.

Your neighbor dentists have often told me you were using, but I paid but little heed thereto, in view of your position as defendant in this, but let it pass by.

Understand me now, however, *very distinctly* to say, that proof of a *single plate made by you after this date*, I will instantly act upon, and lay before the court, demanding an exemplary penalty. Be very careful now, therefore, of your acts, for you are fairly warned, and can ask no consideration.

I advise your calling to see me at this office at the earliest opportunity.

Yours respectfully,
(Signed) JOSIAH BACON, *Treasurer.*

We request of our readers a comparison of this letter, written September 21st, 1870, with the extracts from the contract of agreement with J. B. Newbrough, made July 1st, 1869, in order that they may appreciate how easily Mr. Bacon can NOT tell the truth. S. S. W.

---

SWORN STATEMENT OF DR. B. E. GARDNER. ORIGINAL IN THE POSSESSION OF S. S. WHITE.

*State of New Jersey,*<br>*County of Ocean.* } ss:

I, Benoni E. Gardner, formerly a practicing dentist in Providence, R. I., and now retired from practice, and residing near Bricksburg, Ocean County, New Jersey; do make and execute the following statement to wit: That touching the suit in the First Circuit Court carried on against me, and known as the case of the Goodyear Dental Vulcanite Company, of Boston, and Josiah Bacon *vs* Benoni E. Gardner, I never was and never supposed myself to be a veritable defendant in that suit, nor in the appeal thereof to the Supreme Court of the United States. That I allowed it to be carried on by my friend, Dr. John B. Newbrough, of New York, because I was his licenses. That all the first stages of it were managed and paid for by said Newbrough. *I made the appeal at the request of Mr. Jabez Holmes, acting as agent or attorney of Josiah Bacon,* because he promised me a protection from Bacon, and told me that the appeal was desired by both parties. Dr. Newbrough was spoken of as the other party; I was considered only as nominal defendant. I never paid one dollar costs or expenses, and never considered that I had a dollar of interest in the decision.

I received a paper in writing from Josiah Bacon, Treasurer of the Co., which I considered made me safe from being put to cost or to pay any damages if the suit was decided in their favor. I never had any thought or idea of the reason for the appeal than what Mr. Holmes told me, until recently.

I now think I was deceived about this, and believe that my name has been used to carry this suit ever since Dr. Newbrough settled with the Company, as if I was the defendant, when I was not such in fact. Yet I did not knowingly or willfully consent to the collusion with the knowledge that doing as I did would be aiding the Bacon party to sustain the Cummings patent against the dentists. I say that I supposed the appeal was made to determine whether the Haering patent, owned by Dr. Newbrough, or the Cummings patent, owned by the Goodyear Dental Vulcanite Co., had the best title, and that idea was what was in my mind when I agreed to sign the appeal.

(Signed) BENONI E. GARDNER.

Subscribed and affirmed to before me, a Notary Public for New Jersey, this sixth day of June, 1872, at Bricksburg, N. J.

(Signed) ALBERT M. BRADSHAW,<br>*Notary Public for New Jersey*

[SEAL.]

[EXTRACT FROM A LETTER.]

BRICKSBURG, JUNE 13th, 1872.

S. S. WHITE:

DEAR SIR, * * * In reply to your question, If I know Henry W. Gardner, I would say that I do not. * * *

(Signed) B. E. GARDNER.

---

LETTER FROM SAMUEL J. GLASSEY

This was the only reply elicited by the note in which we offered to give General Foster five hundred dollars if he won the case on Gardner's appeal:

19 PARK PLACE, NEW YORK, March 20th, 1872.

SAMUEL S. WHITE, ESQ., Philadelphia.

SIR,—The communication which you sent to my friend and partner, General Foster, dated 16th inst., was handed to him, as you doubtless intended it should be, a few minutes before the opening of the Court, and while he was preparing for his argument.

As I am his associate in the case, have acted in it with him since September, 1869, sharing his labors and responsibilities, and participating in his earnings, that letter applies to me as well as to him.

It presents very grave questions, which I do not now propose to argue, but to state. If the statements contained in your letter are all true, and its insinuations justified by the facts, Foster and I are unfit and unworthy to practice our profession. If your statements are not all true, and your insinuations are not justified, you are unfit for, and unworthy of, association with, or recognition by, honest men, in any relation of life.

Which party is right must be determined. You will be invited to attend a conference to be held in this city within a few days, at which a report will be made of what has occurred at Washington. Your letter will be read and commented upon.

I hope you will attend, as I have some remarks to make which I would prefer to make in your presence.

Your obedient Servant,

SAMUEL J. GLASSEY.

"Salt is good, but if the salt have lost his saltness?" ! ! !

We print Mr. Glassey's letter *verbatim* as a *bonne bouche*.

We never made any insinuations. All our statements are now proven irrefutably. To ask Mr. Foster to execute the Junior's judgement would only provoke another "smile," or at most an answer which contained neither confession nor denial.

Mr. Glassey is cooler now than when he wrote the above. If, however, on seeing the proofs, he cries, "*Mea culpa*, 'I am unfit and unworthy to practice our profession,' " who will controvert it?

